# Reduced Cell Excitability of Cardiac Postganglionic Parasympathetic Neurons Correlates With Myocardial Infarction-Induced Fatal Ventricular Arrhythmias in Type 2 Diabetes Mellitus

**DOI:** 10.3389/fnins.2021.721364

**Published:** 2021-08-18

**Authors:** Wenfeng Hu, Dongze Zhang, Huiyin Tu, Yu-Long Li

**Affiliations:** ^1^Department of Emergency Medicine, University of Nebraska Medical Center, Omaha, NE, United States; ^2^Department of Cellular & Integrative Physiology, University of Nebraska Medical Center, Omaha, NE, United States

**Keywords:** action potential, cardiac autonomic neuropathy, cardiac vagal neuron, heart rate variability, diabetes mellitus, myocardial infarction, ventricular arrhythmias

## Abstract

**Objective:**

Withdrawal of cardiac vagal activity is considered as one of the important triggers for acute myocardial infarction (MI)-induced ventricular arrhythmias in type 2 diabetes mellitus (T2DM). Our previous study demonstrated that cell excitability of cardiac parasympathetic postganglionic (CPP) neurons was reduced in T2DM rats. This study investigated whether cell excitability of CPP neurons is associated with cardiac vagal activity and MI-induced ventricular arrhythmias in T2DM rats.

**Methods:**

Rat T2DM was induced by a high-fat diet plus streptozotocin injection. MI-evoked ventricular arrhythmia was achieved by surgical ligation of the left anterior descending coronary artery. Twenty-four-hour, continuous ECG recording was used to quantify ventricular arrhythmic events and heart rate variability (HRV) in conscious rats. The power spectral analysis of HRV was used to evaluate autonomic function. Cell excitability of CPP neurons was measured by the whole-cell patch-clamp technique.

**Results:**

Twenty-four-hour ECG data demonstrated that MI-evoked fatal ventricular arrhythmias are more severe in T2DM rats than that in sham rats. In addition, the Kaplan-Meier analysis demonstrated that the survival rate over 2 weeks after MI is significantly lower in T2DM rats (15% in T2DM+MI) compared to sham rats (75% in sham+MI). The susceptibility to ventricular tachyarrhythmia elicited by programmed electrical stimulation was higher in anesthetized T2DM+MI rats than that in rats with MI or T2DM alone (7.0 ± 0.58 in T2DM+MI group vs. 3.5 ± 0.76 in sham+MI). Moreover, as an index for vagal control of ventricular function, changes of left ventricular systolic pressure (LVSP) and the maximum rate of increase of left ventricular pressure (LV dP/dt_max_) in response to vagal efferent nerve stimulation were blunted in T2DM rats. Furthermore, T2DM increased heterogeneity of ventricular electrical activities and reduced cardiac parasympathetic activity and cell excitability of CPP neurons (current threshold-inducing action potentials being 62 ± 3.3 pA in T2DM rats without MI vs. 27 ± 1.9 pA in sham rats without MI). However, MI did not alter vagal control of the ventricular function and CPP neuronal excitability, although it also induced cardiac autonomic dysfunction and enhanced heterogeneity of ventricular electrical activities.

**Conclusion:**

The reduction of CPP neuron excitability is involved in decreased cardiac vagal function, including cardiac parasympathetic activity and vagal control of ventricular function, which is associated with MI-induced high mortality and malignant ventricular arrhythmias in T2DM.

## Introduction

The global burden of diabetes has dramatically risen over the last two decades, and diabetes is expected to affect more than 700 million adults by 2045 (Saeedi et al., 2019), with most having type 2 diabetes mellitus (T2DM, 90 to 95% of diabetic population) (Souza et al., 2011). The leading cause of mortality and morbidity in patients with T2DM is cardiovascular disease (Bissinger, 2017; Schmidt, 2019), among which acute myocardial infarction (MI)-related ventricular arrhythmia is the major cause of mortality in T2DM (Berry et al., 2007; Tancredi et al., 2015). Patients with T2DM are two to four times more likely to die from MI than non-diabetic patients (Berry et al., 2007; Raghavan et al., 2019). Although well-known therapies, including better glycemic control over time, have been noted in patients with T2DM, clinical studies showed that intensive glucose control failed to reduce MI–related mortality in T2DM patients (Duckworth et al., 2009; Reaven et al., 2019). Therefore, exploring the mechanisms responsible for MI-induced high mortality in T2DM would help to develop novel therapeutic interventions for improving prognosis and reducing the mortality in patients with T2DM.

Withdrawal of cardiac vagal activity, a common complication of T2DM, is associated with arrhythmia-related sudden cardiac death in patients with T2DM (Valensi et al., 2001; Sanya et al., 2003). Cell excitability of cardiac parasympathetic postganglionic (CPP) neurons located in intracardiac ganglia is a pivotal factor for acetylcholine (ACh) release from cardiac vagal nerve terminals and resultant regulation of cardiac function through binding to muscarinic ACh receptors (Armour, 1991; Akiyama and Yamazaki, 2000). A clinical study reported that ACh release from cardiac vagal nerve terminals is attenuated in T2DM patients (Oberhauser et al., 2001). Our previous studies found that cell excitability of CPP neurons is reduced in T2DM rats (Liu et al., 2012). Rat intracardiac ganglia are divided into the sinoatrial ganglion and atrioventricular ganglion (AVG) (Sampaio et al., 2003). Since the ventricle receives projection of nerve terminals from the AVG (Pardini et al., 1987), our previous study further demonstrated that reduced cell excitability of CPP neurons located in AVG could contribute to the withdrawal of ventricular vagal function and ventricular arrhythmogenesis (Zhang et al., 2018). More importantly, using the normal rats, our previous study also found that malignant ventricular arrhythmias mainly occur in the early and late stages after MI, whereas electrical remodeling of CPP neurons highly correlates with the occurrence of ventricular arrhythmias in the setting of an MI-induced chronic heart failure but not acute MI (Zhang et al., 2017). However, it is unclear whether reduced cell excitability of CPP neurons correlates to acute MI-induced malignant ventricular arrhythmias and high mortality in the T2DM state. In the present study, we observed the correlation between cardiac vagal neuron excitability and MI-induced malignant ventricular arrhythmias in T2DM rats. We hypothesized that reduced cell excitability of CPP neurons located in the AVG is involved in the withdrawal of ventricular parasympathetic postganglionic activity, which further aggravates MI-induced ventricular arrhythmias and mortality in the T2DM state.

## Materials and Methods

The authors declare that all data support the finding of this study are available from the responding author on reasonable request. The study conforms to guidelines for the Care and Use of Laboratory Animals and was approved by the Institutional Animal Care and Use Committee (IACUC) at the University of Nebraska Medical Center (approved IACUC number: 18-023-04-FC). For all invasive procedures, buprenorphine (0.05 mg/kg, s.c., Reckitt Benckiser Pharmaceuticals Inc., Richmond, VA, United States) served as an analgesic and was given for three post-operative days. After *in vivo* experiments were performed, rats were euthanized with 0.39 ml/kg of Fatal-Plus euthanasia solution (about 150 mg/kg pentobarbital, i.p., Vortech Pharmaceuticals, Dearborn, MI, United States). Four groups of rats were used in the present study, including sham control, sham+MI, T2DM control, and T2DM+MI.

### T2DM Rat Model

Male Sprague-Dawley rats weighing 180–200 g (6–7 weeks of age, Sasco) were housed two per cage under controlled temperature and humidity and a 12-h:12-h dark/light cycle. Water and rat chow were provided *ad libitum*. The rats were randomly assigned to sham (*n* = 40) and T2DM rats (*n* = 40). T2DM was induced by a combination of high-fat diet and streptozotocin (STZ) treatment as previously described (Liu et al., 2012, 2015), which has been used in many studies (Zhang et al., 2009, Zheng et al., 2014; Coppey et al., 2012; Neuschafer-Rube et al., 2014). First, the rats were fed a high-fat diet consisting of 42% fat, 42.7% carbohydrate, and 15.2% protein (Harlan Teklad adjusted fat diet, Harlan Teklad, Madison, WI) for 4 weeks. Then, the rats were intraperitoneally injected with STZ (30 mg/kg) and continued on the high-fat diet. Fasting blood glucose and body weight in all rats were measured weekly. In the sham group, the rats were fed a normal chow diet consisting of 13% fat, 53% carbohydrate, and 34% protein (Harlan Tekladsterilizable rodent diet; Harlan Teklad, Madison, WI). All experiments were performed at 12–14 weeks of feeding with either the normal chow diet or high-fat diet because our previous study revealed the characteristics of T2DM (hyperlipidemia, insulin resistance, and hyperglycemia) (Liu et al., 2012). Rats receiving a high-fat diet plus streptozotocin with fasting blood glucose < 250 mg/dl (*n* = 8) were excluded from this study. Basal hemodynamic and metabolic characteristics from sham and T2DM rats were summarized in Supplementary Table 1.

### Implantation of the ECG Telemeter and ECG Recording in Conscious Rats

Implantation of the ECG telemeter (Millar Instruments, Houston, TX, United States) was performed as described previously (Opitz et al., 1995; Baltogiannis et al., 2005; Shiba et al., 2012). Briefly, the rat was anesthetized with 2% isoflurane (Butler Schein Animal Health, Dublin, OH, United States). The skin was shaved and sterilized. After a laparotomy was performed at the Linea Alba (abdomen), an ECG telemeter (TRM54PB, Kaha Science, Auckland, New Zealand) was placed into the abdominal cavity and secured to the abdominal wall at the best position for battery recharging and signal communication. In accordance with the Millar User Manual, the bipolar electrodes were tunneled subcutaneously for ECG recording. The negative electrode was secured in the upper sternal midline, and the positive electrode was attached to the underlying tissue near the left side of the xiphoid process. To reduce the electrical noise during the recording, the electrodes were kept together and run alongside one another as far as practical. All incisions were sutured in two layers. An ECG recording was performed one week after surgery.

After implantation of the ECG telemeter, the rat was placed on a SmartPad receiver (Millar Instruments, Houston, TX, United States). For quantification of ventricular arrhythmic events, 24-h continuous ECG signals were acquired in unrestrained, conscious rats. Real-time ECG signals were digitalized and analyzed by PowerLab 8/30 Data Acquisition System with LabChart 8 software and ECG analysis module (AD Instruments, Colorado Springs, CO, United States). The number of premature ventricular contractions (PVCs) and the cumulative duration of ventricular tachycardia/fibrillation (VT/VF) were manually counted during 24-h continuous ECG recording. VT was defined as PVCs lasting ≥4 beats. VF was defined as rapid, irregular QRS complexes.

### Measurement of Heart Rate Variability and Ventricular Electrical Activity in Conscious Rats

Heart Rate Variability (HRV) measurement is a widely used approach for the determination of autonomic function in T2DM patients in the clinic (Benichou et al., 2018; Akinlade et al., 2021). For quantification of HRV, 24-h continuous ECG signals were acquired in unrestrained, conscious rats. Real-time ECG signals were digitalized by PowerLab 8/30 Data Acquisition System with LabChart 8 software and HRV analysis module (AD Instruments, Colorado Springs, CO, United States). Heart Rate Variability was analyzed and averaged from ECG segments during the 24-h recording in conscious rats. Heart Rate Variability analysis including low-frequency power (LF) from 0.2 to 0.75 Hz, high-frequency power (HF) from 0.75 to 2.5 Hz, and LF/HF ratio was performed in the current study (Murakami et al., 2014; Rossi et al., 2014; Carnevali et al., 2015).

To quantify the ventricular electrical activity, ventricular arrhythmogenesis-related ECG markers including QT and corrected QT (QTc) intervals, as well as dispersions, were calculated from ECG recording in conscious rats. QTc interval was calculated by Bazett’s formula (QT/$\sqrt{RR}$, where RR is RR interval) (Costa et al., 2008). As an index of the spatial dispersion of the ventricular repolarization, QT and QTc dispersions were calculated by the equations: QT dispersion = QT_max_ – QT_min_ and QTc dispersion = QTc_max_ – QTc_min_, where QT_max_ and QTc_max_ are the maximum QT interval and the maximum QTc interval; QT_min_ and QTc_min_ are the minimum QT interval and the minimum QTc interval. T-peak to T-end (Tpe) interval, another marker of transmural dispersion of the ventricular repolarization, was calculated and served as an ECG marker of ventricular arrhythmia (Yan et al., 2003; Antzelevitch, 2007; Yagishita et al., 2015).

### Measurement of Acute Myocardial Infarction-Evoked Ventricular Arrhythmias and Mortality in Conscious Rats

Since MI-related ventricular arrhythmia is the most common cause of mortality in T2DM patients (Berry et al., 2007; Tancredi et al., 2015), MI achieved by the ligation of left anterior descending coronary artery (LAD) was used to induce the fatal ventricular arrhythmia. Briefly, rats were anesthetized with 2% isoflurane (Butler Schein Animal Health, Dublin, OH, United States) for LAD ligation, and control rats, including sham control and T2DM control, underwent the same surgery without LAD ligation. Continuous 24-h ECG recording was started after the animal woke up from the surgery of LAD ligation. Incidence and duration of VT/VF were quantified within 24 h after MI. The Kaplan-Meier analysis was used for estimation of survival rate over 2 weeks in all groups because previous studies (Nakamura et al., 2004; Onai et al., 2007) showed that animal death induced by acute MI usually occurs during the first 2 weeks of MI.

### Measurements of Inducibility of Ventricular Arrhythmia

After the observation of survival rate, the remaining rats were anesthetized with 800 mg/kg urethane combined with 40 mg/kg α-chloralose (i.p.). The trachea was cannulated to facilitate mechanical respiration. The animal’s body temperature was maintained at 37°C with an animal temperature controller (ATC 1000; World Precision Instruments, Sarasota, FL, United States). The right femoral vein was cannulated with a polyethylene-50 catheter for the administration of saline. The left femoral artery was cannulated with a polyethylene-50 catheter for blood pressure and heart rate monitoring. Surface lead-II ECG was recorded using subcutaneous electrodes connected to a biological amplifier (AD Instruments, Colorado Springs, CO, United States). Hemodynamic data (Table 1) were recorded by PowerLab 8/30 Data Acquisition System with LabChart 8 software (AD Instruments, Colorado Springs, CO, United States). Then, a left thoracotomy was performed in the fourth intercostal space. After the heart was exposed, the pericardium was carefully removed. A bipolar platinum stimulating electrode was placed on the right ventricular outflow tract for electrical stimulation (Kang et al., 2009). Programmed electrical stimulation (PES) was performed by a programmed electrical stimulator (Digital Pulse Generator 1831; WPI, United States) and an isolator (A320R Isostim Stimulator; WPI, United States). The pulse current output was set to twice the capture threshold and a 2-ms pulse width. After measurement of the ventricular effective refractory period (Gui et al., 2013), a programmed stimulation protocol combined by single (S2), double (S3), or triple extra- stimulus (S4) after a train of eight stimuli (8 × S1) was designed to induce ventricular tachyarrhythmia as described previously (Kang et al., 2009; Shiba et al., 2012; Hong et al., 2014; Zhang et al., 2021a, b). Ventricular tachyarrhythmia was considered as non-inducible when either PES failed to induce ventricular premature beats or self-terminated ventricular premature beats <6. Ventricular tachyarrhythmia was considered as non-sustained when it lasted ≤15 beats and sustained when it lasted >15 beats before spontaneously terminating (Belichard et al., 1994; Nguyen et al., 1998). Inducibility of ventricular tachyarrhythmia was quantified by a quotient of ventricular arrhythmia score as described previously (Nguyen et al., 1998; Kang et al., 2009; Zhang et al., 2021a, b).

**TABLE 1:** Hemodynamic and morphological characteristics in all groups of rats.

	Sham		T2DM	
	Control (*n* = 6)	MI (*n* = 6)	Control (*n* = 6)	MI (*n* = 3)
MBP (mmHg)	103.7 ± 4.0	105.5 ± 4.3	100.5 ± 4.3	106.3 ± 9.3
HR (bpm)	352.8 ± 10.7	367.8 ± 10.0	353.7 ± 12.3	373.3 ± 20.3
LVSP (mmHg)	122.3 ± 3.3	113.8 ± 3.7	118.2 ± 3.0	110.7 ± 7.1
LVEDP (mmHg)	1.9 ± 0.3	16.7 ± 0.7*	1.9 ± 0.2	15.3 ± 7.1^†^
LV dP/dt_max_ (mmHg/s)	6095.2 ± 237.8	5578.8 ± 201.0	5877.8 ± 251.3	5194.3 ± 356.8
Infarct size (% of LV)	N/A	47.8 ± 1.4*	N/A	48.3 ± 2.6^†^

MBP, mean blood pressure; HR, heart rate; LV, left ventricle; LVSP, left ventricular end-systolic pressure; LVEDP, left ventricular end-diastolic pressure; LV dP/dt_max_, the maximum rate of increase of left ventricular pressure. Data are means ± SEM. Statistical significance was determined by one-way ANOVA with post-hoc Bonferroni test. *P < 0.05 vs. Sham Control; †p < 0.05 vs. T2DM Control.

### Measurements of Vagal Control of Ventricular Function

After the measurement of the inducibility of ventricular arrhythmia, vagal control of ventricular function was determined, as described previously (Zhang et al., 2017, 2018). Briefly, a Millar pressure transducer (SPR 524; size, 3.5-Fr; Millar Instruments, Houston, TX, United States) was slowly inserted into the right carotid artery and carefully advanced to the left ventricle for measurement of left ventricular systolic pressure (LVSP) and the maximum rate of increase of left ventricular pressure (LV dP/dt_max_). Then, bilateral cervical vagal nerves, sympathetic nerves, and aortic depressor nerves (an afferent branch of the vagal nerve innervating the aortic arch and thoracic aorta) were isolated and transected to avoid the influence of the arterial baroreflex. The peripheral end of the left vagal nerve was placed on a bipolar stimulating electrode for vagal efferent nerve stimulation. Left vagal efferent nerve stimulation was applied by a Grass S9 stimulator (Grass instruments, Quincy, MA, United States) with 10 s of constant-frequency stimulation (0.1 ms pulse duration and intensity of 7.5 V at 1-100 Hz). As the index of vagal control of ventricular function, changes of LVSP and LV dP/dt_max_ in response to different frequencies of left vagal efferent nerve stimulation were recorded by PowerLab 8/30 data acquisition system with LabChart 8 software (ADInstruments, Colorado Springs, CO, United States). After *in vivo* experiments were done, the rat heart was removed to measure infarct size, which was determined using a colorimetric technique coupled to a computerized planimetric analysis (Adobe Photoshop CS5 Extended). The percentage of infarct area to the whole left ventricle was quantified using Adobe Photoshop CS5 Extended (Adobe Systems Incorporated, CA).

### Labeling, Isolation of AVG Neurons, and Whole Cell Patch-Clamp Recording of Action Potentials (APs)

Since the ventricular myocardium receives projection of nerve terminals from the AVG (Pardini et al., 1987), it is possible that the AVG also innervates other parts of the heart. To explore the relationship between vagal activity and ventricular arrhythmia, we used a transported fluorescent dye (red color DiI) to retrograde-label AVG neurons projecting to the ventricular myocardium. Under anesthetized condition (800 mg/kg urethane combined with 40 mg/kg α-chloralose, i.p.), a left thoracotomy was performed in the fourth intercostal space. Eight injections (2 μl DiI for each injection) were made subepicardially into the left ventricle, using a fine-tipped glass micropipette connected to a microinjector (Nanoliter 2000, WPI, Sarasota, FL, United States). The surgical incision was closed, and terminal experiments were performed at 2 weeks after surgery to allow the dye to diffuse to the neurons.

At the terminal experiment, the AVG located in a white epicardial adipose pad at the junction of inferior pulmonary veins and left atrium was exposed. AVG neurons were isolated by a 2-step enzymatic digestion protocol, as described previously (Liu et al., 2012; Tu et al., 2014; Zhang et al., 2017, 2018). Isolated AVG was placed in ice-cold modified Tyrode solution (mmol/L): 140 NaCl, 5 KCl, 10 HEPES, and 5 glucose. The AVG was minced with microscissors and incubated with a modified Tyrode solution containing 0.1% collagenase and 0.1% trypsin for 30 min at 37°C. The tissue was then transferred to a modified Tyrode solution containing 0.2% collagenase and 0.5% bovine serum albumin for 30 min of incubation at 37°C. The isolated neurons were cultured at 37°C in a humidified atmosphere of 95% air-5% CO_2_for patch-clamp experiments, as described previously (Liu et al., 2012; Tu et al., 2014; Zhang et al., 2017, 2018). Briefly, APs were recorded only in DiI-labeled AVG neurons (i.e., CPP neurons) by the whole-cell patch-clamp technique using Axopatch 200B patch-clamp amplifier (Axon Instruments). In current-clamp experiments, AP was elicited by a ramp current injection of 0-100 pA, and the current threshold-inducing AP or threshold potential was measured at the beginning of the 1^st^ AP. The frequency of APs was measured in a 1-sec current clamp. The patch pipette solution was composed of (in mM): 105 K-aspartate, 20 KCl, 1 CaCl_2_, 5 MgATP, 10 HEPES, 10 EGTA, and 25 glucose (pH 7.2; 320 mOsm/L). The bath solution was composed of (in mM): 140 NaCl, 5.4 KCl, 0.5 MgCl_2_, 2.5 CaCl_2_, 5.5 HEPES, 11 glucose, and 10 sucrose (pH 7.4; 330 mOsm/L). The junction potential was calculated to be +12.3 mV, and the membrane potential was corrected using this value. P-clamp 10.2 program (Axon Instruments) was used for data acquisition and analysis. All experiments were performed at room temperature (22–24°C).

### Statistical Analysis

All data are presented as means ± SEM. SigmaPlot 12 was used for data analysis. Statistical significance was determined by one-way ANOVA with post-hoc Bonferroni test for multi-group comparison. A student’s unpaired *t*-test was used to perform a two-group comparison. A Chi-Square test was used to analyze the incidence of ventricular arrhythmias. The significance in survival rate was determined by Kaplan-Meier Survival Analysis with Log-Rank test. Normal distribution of data was confirmed with Kolmogorov-Smirnov test and equal variance with Levene’s test. Statistical significance was accepted when *p* < 0.05.

## Results

### MI-Related Mortality and Fatal Ventricular Arrhythmias in Conscious Sham and T2DM Rats

**FIGURE 1:**
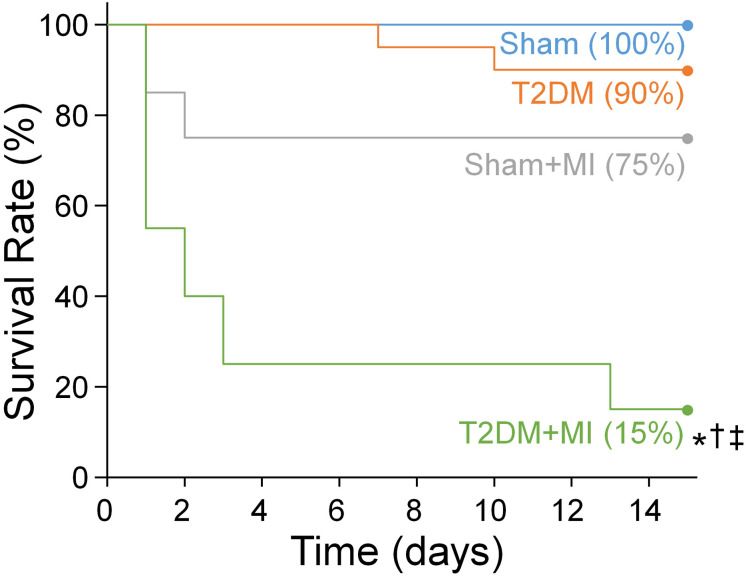
The Kaplan-Meier analysis of survival rate within 2 weeks after MI in all experimental groups. There was no death occurred in sham rats and 90% of T2DM rats without MI survived during the 2-week observational period. The survival rate within 2 weeks after MI was significant lower in T2DM rats (15% in T2DM+MI), compared with sham rats (75% survival rate in sham+MI). *n* = 20 rats per group. Statistical significance was determined by Log-Rank test. *P < 0.05 vs. sham; ^†^*p* < 0.05 vs. T2DM; ^‡^*p* < 0.05 vs. sham+MI.

Since MI-related ventricular arrhythmia is the most common cause of mortality in T2DM patients (Berry et al., 2007; Tancredi et al., 2015), MI achieved by LAD ligation was used to induce the fatal ventricular arrhythmia, and the survival rate over 2 weeks after MI was quantified in conscious rats. Figure 1 shows the collected data relative to the Kaplan-Meier survival curves for these experimental groups. The Kaplan-Meier survival analysis demonstrated that no death occurred in sham rats, and 90% (18 out of 20) of T2DM rats without MI survived during the 2-week observational period. The survival rate within 2 weeks after MI was significantly lower in T2DM rats than that in sham rats (15% in T2DM+MI group vs. 75% in sham+MI group, *p* < 0.05).

**FIGURE 2:**
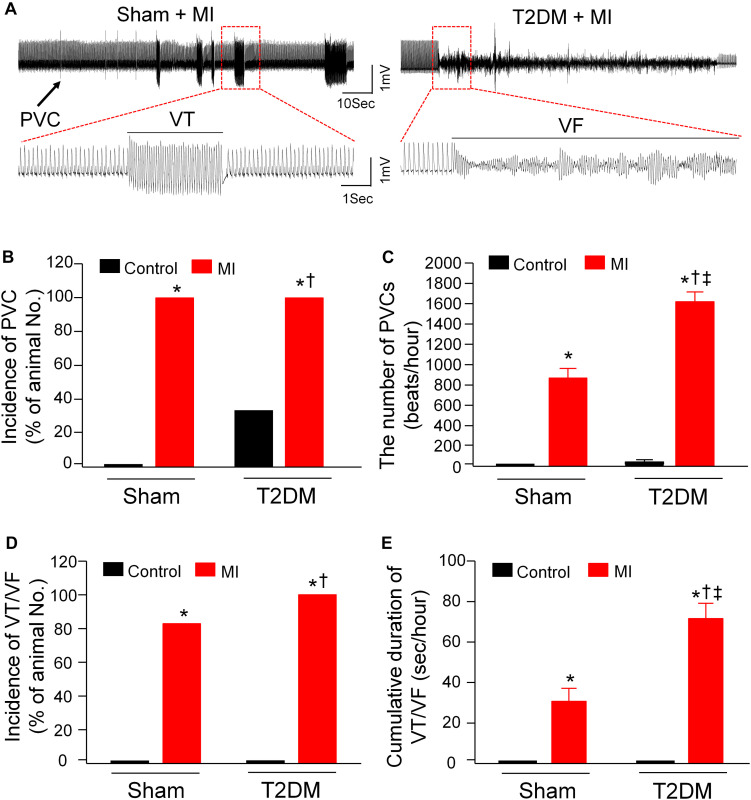
Spontaneous ventricular arrhythmias in all experimental groups of conscious rats. **(A)**: Raw ECG recordings for premature ventricular contractions (PVCs) and ventricular tachycardia/fibrillation (VT/VF) in conscious sham and T2DM rats within 24 h after MI. **(B–E)**: Mean data for incidence of PVCs (B), number of PVCs **(C)**, incidence of VT/VF **(D)**, and cumulative duration of VT/VF **(E)** in all groups of conscious rats. No PVCs and VT/VF were observed in sham rats, whereas a few PVCs but not VT/VF were detected in some T2DM rats without MI. MI induced significant increases in the number of PVCs and the cumulative duration of VT/VF in T2DM rats, compared with sham+MI. Statistical significance was determined by a Chi-Square test for data presented in panel **(B)** and **(D)**. Statistical significance was determined by one-way ANOVA with post-hoc Bonferroni test for data presented in panel **(C,E)**. Data are means ± SEM; *n* = 6 rats per group.*P < 0.05 vs. sham; ^†^*p* < 0.05 vs. T2DM; ^‡^*p* < 0.05 vs. sham+MI.

Considering that MI-related malignant ventricular arrhythmia is the most major cause of mortality in T2DM (Berry et al., 2007; Tancredi et al., 2015), ventricular arrhythmic events, monitored by 24-h, continuous ECG recording in conscious animals, were assessed in all experimental groups (Figure 2). In sham rats, no PVCs and VT/VF were observed. There were a few PVCs that were detected in some T2DM rats without MI (2 out of 6 rats, Figure 2). MI significantly induced malignant ventricular arrhythmias (VT/VF) in sham (5 out of 6 rats) and T2DM rats (6 out of 6 rats, Figure 2). More importantly, the incidence and cumulative duration of VT/VF induced by MI were much higher/longer in T2DM rats than those in sham rats (Figures 2D,E).

### Susceptibility to Ventricular Arrhythmias in All Experimental Groups of Anesthetized Rats

**FIGURE 3:**
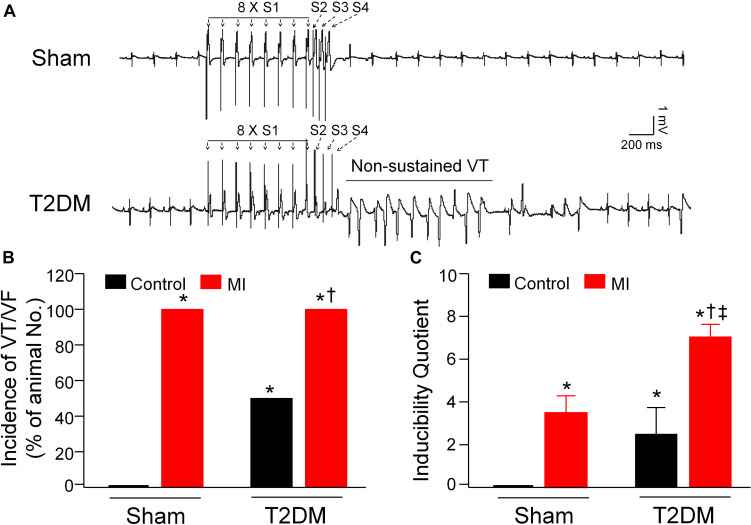
Susceptibility to ventricular arrhythmias determined by programmed electrical stimulation (PES) in all experimental groups of anesthetized rats. **(A)**: Raw data for PES-evoked ventricular tachycardia/fibrillation (VT/VF) in anesthetized sham and T2DM rats. **(B,C)**: Mean data for incidence **(B)** and inducibility quotient **(C)** of PES-evoked VT/VF in all groups of anesthetized rats. Compared with sham rats, a high incidence of VT/VF and inducibility quotient were found in T2DM rats without MI. Both incidence of VT/VF and inducibility quotient were further increased in T2DM rats after MI, compared with sham rats with MI. Data are means ± SEM; *n* = 3–6 rats per group. Statistical significance was determined by one-way ANOVA with post-hoc Bonferroni test. *P < 0.05 vs. sham; ^†^*p* < 0.05 vs. T2DM; ^‡^*p* < 0.05 vs. sham+MI.

Besides the measurement of spontaneous ventricular arrhythmias in conscious rats, PES-triggered inducibility of ventricular arrhythmias (including the incidence of VT/VF and inducibility quotient) in anesthetized rats was also measured to assess the susceptibility to ventricular arrhythmias in all experimental groups (Figure 3). Programmed electrical stimulation did not elicit the occurrence of VT/VF, and the inducibility quotient was zero in sham rats. Compared to sham rats, the inducibility of ventricular arrhythmias, demonstrated by the incidence of VT/VF (6 out of 6 rats) and inducibility quotient (2.5 ± 1.23), was high in T2DM rats without MI. Additionally, the susceptibility to ventricular arrhythmias after MI was higher in T2DM rats than that in sham rats (inducibility quotient 7.0 ± 0.58 in T2DM+MI group vs. 3.5 ± 0.76 in sham+MI group, *p* < 0.05, Figure 3C).

### ECG Analysis of Ventricular Electrical Activity in All Experimental Groups of Conscious Rats

**FIGURE 4:**
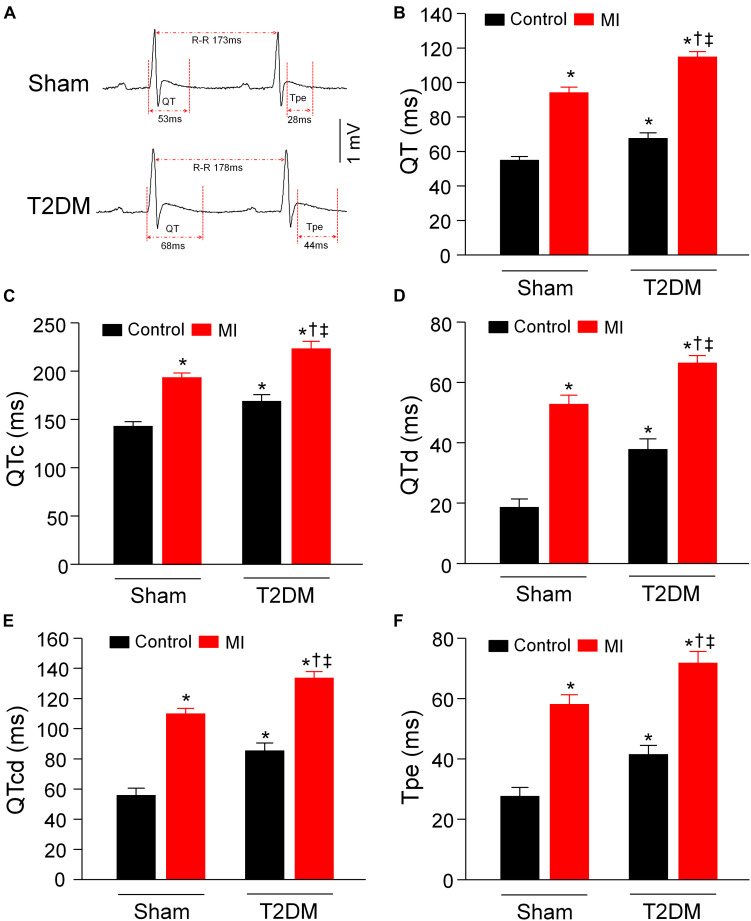
Heterogeneity of ventricular electrical activities calculated from 24-h ECG recording in all experimental groups of conscious rats. **(A)**: Representative tracings for QT and Tpe intervals in sham (upper) and T2DM (lower) rats. **(B–F)**: Quantitative data for QT interval **(B)**, QTc interval **(C)**, QT dispersion **(D)**, QTc dispersion **(E)**, and Tpe **(F)** in all experimental groups. T2DM significantly increased the heterogeneity of ventricular electrical activities, compared to sham rats without MI. MI-induced alterations in the heterogeneity of ventricular electrical activities were more obvious in T2DM rats, compared with sham+MI. Data are means ± SEM; *n* = 6 rats per group. Statistical significance was determined by one-way ANOVA with post-hoc Bonferroni test. *P < 0.05 vs. sham; ^†^*p* < 0.05 vs. T2DM; ^‡^*p* < 0.05 vs. sham+MI.

It has been known that the heterogeneity of ventricular electrical activities (including QT and QTc intervals, QT and QTc dispersions, and Tpe interval) is a critical factor of ventricular arrhythmogenesis (Yagishita et al., 2015). Our data from 24-h radiotelemetry ECG recording in conscious rats demonstrated that T2DM significantly prolonged QT and QTc intervals, QT and QTc dispersions, and Tpe interval, compared to age-matched sham rats (Figure 4). Additionally, our study also demonstrated that acute MI caused the prolongations in QT and QTc intervals, QT and QTc dispersions, and Tpe interval in both sham and T2DM rats, which is consistent with results from clinical studies that acute MI induces a significant prolongation of QT interval (Taylor et al., 1981; Ahnve, 1985; Kenigsberg et al., 2007). However, MI-induced alterations in these ventricular electrical activities were longer in T2DM rats (T2DM+MI group) than those in sham rats (sham+MI group) (Figures 4B–F).

### Power Spectral Analysis of HRV in All Experimental Groups of Conscious Rats

**FIGURE 5:**
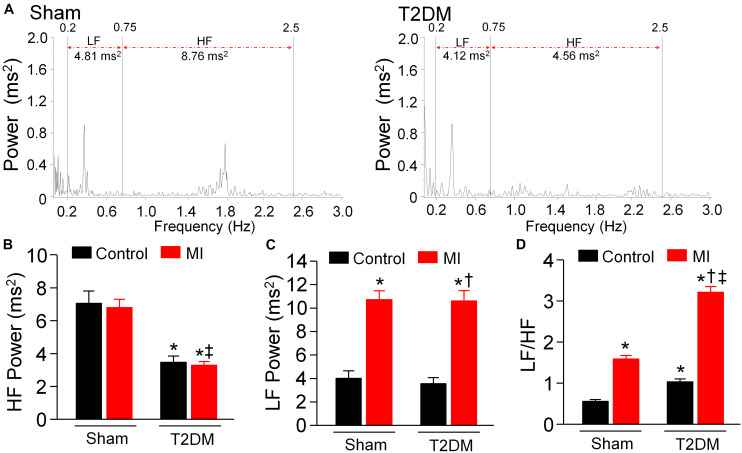
Cardiac autonomic function measured by heart rate variability (HRV) in all experimental groups of conscious rats. **(A)**: Representative data showing HRV measured in sham (left) and T2DM (right) rats. Spectral power was quantified for low-frequency power (LF) from 0.2 to 0.75 Hz and high-frequency power (HF) from 0.75 to 2.5 Hz. **(B–D)**: Quantitative data of HF **(B)**, LF **(C)**, and LF/HF ratio **(D)** from all groups of rats. T2DM reduced HF power. MI increased LF power and LF/HF ratio in sham (sham+MI) and T2DM (T2DM+MI) rats. MI-increased LF/HF ratio was higher in T2DM (T2SM+MI) rats than that in sham (sham+MI) rats. Data are means ± SEM; *n* = 6 rats per group. Statistical significance was determined by one-way ANOVA with post-hoc Bonferroni test. *P < 0.05 vs. sham; ^†^*p* < 0.05 vs. T2DM; ^‡^*p* < 0.05 vs. sham+MI.

Cardiovascular autonomic dysfunction including sympathetic and parasympathetic activities is a common complication of patients with T2DM (Valensi et al., 2001; Sanya et al., 2003; Pop-Busui, 2010). Therefore, we used power spectral analysis of HRV from a 24-h ECG recording to evaluate cardiac autonomic activities in conscious rats. In the power spectral analysis of HRV, HF power comprises cardiac parasympathetic oscillation, while LF power and LF/HF ratio represent cardiac sympathetic activation (Thayer et al., 2010). As an index of cardiac parasympathetic activation, HF power was reduced in T2DM rats without MI (Figures 5A,B), whereas MI did not alter HF power in sham (sham+MI) and T2DM (T2DM+MI) rats (Figure 5B). About cardiac sympathetic activation in all experimental groups, T2DM without MI did not change LF power (Figures 5A,C) and slightly induced a statistically significant increase in LF/HF ratio, compared to the sham group. However, MI significantly increased LF power and LF/HF ratio in sham (sham+MI) and T2DM (T2DM+MI) rats (Figures 5C,D). In particular, MI-increased LF/HF ratio was markedly higher in T2DM (T2DM+MI) rats than that in sham (sham+MI) rats (Figure 5D).

### Ventricular Vagal Function in All Experimental Groups of Anesthetized Rats

**FIGURE 6:**
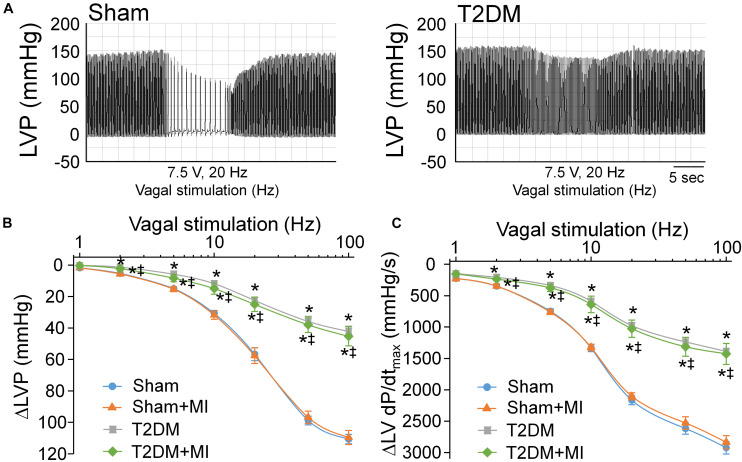
Vagal control of ventricular function determined by changes of left ventricular systolic pressure (LVSP) and the maximum rate of increase of left ventricular pressure (LV dP/dt_max_) in response to different frequencies of left vagal efferent nerve stimulation in all experimental groups of anesthetized rats. **(A)**: Representative recordings demonstrating change of LVSP in response to 7.5 V, 20 Hz of left vagal efferent nerve stimulation in sham and T2DM rats. **(B,C)**: Quantitative data for changes of LVSP **(B)** and LV dP/dt_max_ **(C)** in response to different frequencies (1–100 Hz) of left vagal efferent nerve stimulation in all groups of rats. T2DM blunted the vagal control of ventricular function, as demonstrated by reduced changes of LVSP and LV dP/dtmax in response to vagal efferent nerve stimulation. MI had no effect on vagal control of ventricular function in both sham and T2DM rats. Data are means ± SEM; *n* = 3–6 rats per group. Statistical significance was determined by one-way ANOVA with post-hoc Bonferroni test. *P < 0.05 vs. sham; ^‡^*p* < 0.05 vs. sham+MI.

Activation of the vagal efferent nerve results in a negative inotropic effect in the ventricle, which serves as an index of ventricular vagal function (Lewis et al., 2001). Comparing with age-matched sham rats, changes of LVSP and LV dP/dt_max_ in response to vagal efferent nerve stimulation were blunted in T2DM rats (Figure 6), which demonstrated that vagal control in the ventricle was impaired in the T2DM state. Nevertheless, MI did not affect vagal control in the ventricle in both sham (sham+MI) and T2DM (T2DM+MI) rats (Figure 6).

### Cell Excitability of Isolated CPP Neurons in All Experimental Groups of Rats

**FIGURE 7:**
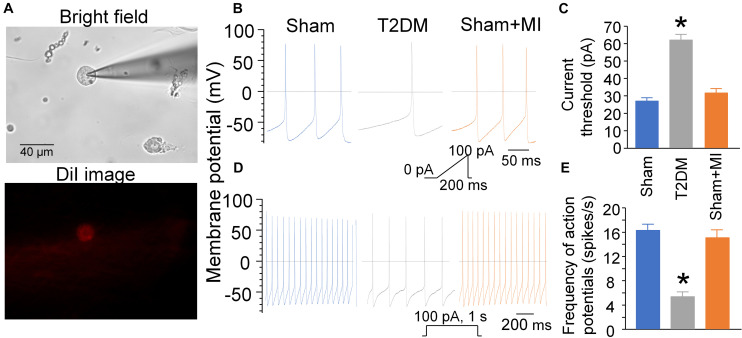
Cell excitability of CPP neurons determined by whole-cell patch-clamp. **(A)**: DiI-labeled AVG neuron (CPP neuron) with red color in fluorescent light. **(B–E)**: Current threshold-inducing action potential (AP, **B,C**) and frequency of AP **(D,E)** in CPP neurons (DiI-labeled AVG neurons) from sham, T2DM, and acute MI rats. Current threshold-inducing APs was calculated at the beginning of the first action potential elicited by a ramp current injection of 0–100 pA during 200 ms. The frequency of APs was measured in a 1-s current clamp with a current injection of 100 pA.T2DM significantly reduced the cell excitability of CPP neurons, as evidenced by increased current threshold-inducing APs and reduced frequency of APs in T2DM rats compared to sham rats. MI had no effects on current threshold-inducing APs and frequency of APs. Data are mean ± SEM; *n* = 10 neurons from 5 rats per group. Statistical significance was determined by one-way ANOVA with post-hoc Bonferroni test. *p < 0.05 vs. sham.

Using a whole-cell patch-clamp technique, we measured current threshold-inducing APs and frequency of APs to determine cell excitability of DiI-labeled AVG neurons (i.e., CPP neurons) (Figure 7). The current threshold-inducing APs was enhanced, and the frequency of APs was reduced in CPP neurons from T2DM rats without MI, compared to that from sham rats (62 ± 3.3 pA and 5 ± 1 spikes/s in T2DM rats without MI vs. 27 ± 1.9 pA and 16 ± 1 spikes/s in sham rats, *P* < 0.05, Figure 7), which is consistent with data from our previous study (Liu et al., 2012). Additionally, T2DM without MI also reduced the maximum rate of depolarization of APs (V_max_) and elongated AP duration at 90% repolarization (APD_90_), whereas it did not affect resting membrane potential (RMP) and overshoot of APs, compared to the sham group of rats (Supplementary Table 2). Moreover, MI did not change current threshold-inducing APs, frequency of APs, RMP, V_max_, overshoot of APs, and APD_90_ in CPP neurons from sham (sham+MI) rats (Figure 7 and Supplementary Table 2) and T2DM (T2DM+MI) rats (data not shown).

## Discussion

From the present study, we further understood why the mortality rate from MI in patients with T2DM is higher than that in non-diabetic patients. In this current study, MI-caused mortality and durations of VT/VF in a state of consciousness are higher/longer in T2DM rats than those in sham rats (Figures 1,2). Compared with sham plus MI rats, susceptibility to ventricular tachyarrhythmia, demonstrated by PES-triggered VT/VF and inducibility quotient in anesthetized rats, is higher in T2DM plus MI rats (Figure 3). Most importantly, T2DM reduced cardiac parasympathetic activation and cell excitability of CPP neurons, whereas MI did not affect cardiac vagal function and CPP neuronal excitability. Therefore, our present study demonstrated for the first time that T2DM-reduced CPP neuronal excitability is a prerequisite factor for MI-related malignant ventricular arrhythmias and high mortality rate in the T2DM state through a reduction of cardiac parasympathetic activation.

Growing evidence demonstrates that cardiac autonomic neuropathy (CAN) is a serious complication in T2DM patients and plays a critical impact on cardiovascular events in diabetic populations (Pop-Busui, 2010). The pathogenesis of CAN is due to the structural and functional impairments in cardiac autonomic neurons that innervate the heart and results in abnormalities in the autonomic control of the heart (Vinik and Ziegler, 2007; Akinlade et al., 2021). To clarify the relationship between CAN and cardiovascular mortality in the T2DM state, it is important to determine both cardiac sympathetic and parasympathetic neuronal functions. Heart rate variability analysis is the most commonly used method and is recommended by Toronto Consensus Panel on the diagnosis of cardiac autonomic neuropathy in patients with T2DM (Benichou et al., 2018; Akinlade et al., 2021). Therefore, it was employed to assess cardiac autonomic function in our present study, in which LF power and LF/HF ratio show cardiac sympathetic activation, and HF power represents cardiac parasympathetic activation. In our current study, T2DM did not induce a significant alteration in cardiac sympathetic activation, as evidenced by no change in LF power and a slight increase in LF/HF ratio in T2DM rats, compared with age-matched sham rats (Figures 5C,D). Some previous studies reported cardiac sympathetic overactivation in Zucker diabetic fatty rats (Thaung et al., 2015) and type 2 diabetes mellitus (Huggett et al., 2003), detected by direct recording of cardiac postganglionic sympathetic nerve activity. In our current study, no significant alteration in cardiac sympathetic tone in T2DM rats might be explained by T2DM-impaired post-synaptic function such as cardiac adrenergic beta-receptor responsiveness (Thaung et al., 2015). Indeed, it remains controversial whether cardiac sympathetic activity is overexcited in T2DM patients. It has been reported that cardiovascular mortality in patients with diabetes is further augmented by diminished sympathetic activity in the presence of CAN (Stevens et al., 1998a). Given that the value of LF power and LF/HF ratio in HRV analysis depends on numerous factors such as cardiac adrenergic beta-receptor sensitivity, post-receptor transduction, and parasympathetic modulation, etc. (Notarius and Floras, 2001), direct recording of cardiac sympathetic nerve activity and measurement of post-synaptic elements such as cardiac sympathetic nerve innervation and adrenergic beta-receptor responsiveness need to be done in future studies to determine the sympathetic control of heart in the T2DM state.

In comparison with cardiac sympathetic neuronal function, cardiac parasympathetic (vagal) neuronal function was reported to be severely affected in patients with T2DM (Freccero et al., 2004). A clinical study demonstrated that the vagal predominance is significantly impaired in proportion to a withdrawal of total autonomic activity (Oida et al., 1999). In agreement with results from clinical findings that cardiac vagal activity is blunted in patients with T2DM, our present data clearly confirmed that cardiac parasympathetic activity (Figure 5B) and vagal control of cardiac function (Figure 6) were markedly reduced in T2DM rats, suggesting that the withdrawal of cardiac vagal activity is the hallmark of T2DM. Previous studies found that cardiac vagal dysfunction is associated with ventricular arrhythmia-related sudden cardiac death in T2DM patients (Valensi et al., 2001; Sanya et al., 2003). In our present study, acute MI significantly increased mortality rate, elongated durations of VT/VF, and enhanced susceptibility to VT/VF in T2DM (T2DM+MI) rats, compared with sham rats with the same MI procedure. An interesting phenomenon in the present study is that MI-induced cardiac sympathetic overactivation was higher in T2DM+MI rats than that in sham+MI rats (Figure 5D) when MI did not affect cardiac parasympathetic activation in sham and T2DM rats (Figure 5B). Much evidence has demonstrated the interactive inhibitions between cardiac sympathetic and parasympathetic nervous systems at multiple levels, including peripheral ganglia and nerve terminals innervated the heart (Armour and Butler, 1989; Levy, 1990; Lameris et al., 2000). Therefore, a reasonable explanation for the above phenomenon is that T2DM-induced withdrawal of cardiac vagal activity precipitates a further increase in MI-triggered cardiac sympathetic overactivation in T2DM+MI rats, compared to sham+MI rats. From these data, we assume that T2DM creates a prerequisite condition, the withdrawal of cardiac vagal activity, to raise the myocardial susceptibility to ventricular arrhythmias for MI-related fatal ventricular arrhythmias and high mortality rate in the T2DM state, although T2DM alone does not induce spontaneous ventricular arrhythmias without high mortality rate.

Additionally, while acute MI induces significant changes in cardiac autonomic function and heterogeneity of ventricular electrical activities, it does not affect the vagal control of ventricular function and CPP neuronal excitability. The vagal control of ventricular function and CPP neuronal excitability are reduced in the T2DM state. However, acute MI fails to further reduce the vagal control of ventricular function and CPP neuronal excitability in T2DM rats, as evidenced in Figures 6, 7. Although MI-induced chronic heart failure causes a cardiac parasympathetic withdrawal due to the impaired firing frequency (Vaseghi et al., 2017) or neuronal hypertrophy (Singh et al., 2013) of the cardiac parasympathetic neurons, it remains unclear if there is more impairment in cardiac vagal function and CPP neuronal excitability in the T2DM state with MI-induced chronic heart failure. Given that the electrical remodeling of CPP neurons and vagal control of ventricular function begin to decrease in 8 weeks after MI, and appear significant decreases in 14 weeks after MI (Zhang et al., 2017), it might expect that the vagal control of ventricular function and CPP neuronal excitability would be reduced more in T2DM with MI-induced chronic heart failure.

Our previous study demonstrated that reduced cell excitability of cardiac vagal neurons contributes to cardiac vagal dysfunction and ventricular arrhythmogenesis (Zhang et al., 2018). Combining with the results in the present study, we consider that the low excitability of CPP neurons might be involved in the withdrawal of cardiac vagal activity and further associated with MI-related malignant ventricular arrhythmias and high mortality rate in the T2DM state. Despite the withdrawal of cardiac vagal activity in T2DM that was illustrated in the present study, the underlying mechanisms responsible for T2DM-impaired cardiac vagal function remain unclear. Beyond T2DM-reduced CPP neuron excitability (Figure 7) and ACh release from cardiac vagal nerve terminals (Oberhauser et al., 2001), we believe that other components such as alterations in post-synaptic elements also play a critical role in the withdrawal of cardiac vagal activity in the T2DM state. Unlike the traditional principle that cardiac vagal nerves slow sinus rate and atrioventricular conduction with less influence on the ventricle (Sroka, 2004; Brack et al., 2013), advanced techniques have affirmed dense vagal innervation in the ventricle from many species, including rat (Xu et al., 2006) and human (Pauza et al., 2000). Although cardiac sympathetic denervation has been evidenced by many studies involving T2DM patients (Koistinen et al., 1996; Stevens et al., 1998b), the innervation status of cardiac vagal nerve terminals remains unexplored in the T2DM state. Further studies are needed to determine if the innervation status of cardiac vagal nerve terminals contributes to MI-related fatal ventricular arrhythmias in the T2DM state.

From our present study, we cannot clearly know what is involved in the low excitability of CPP neurons in T2DM. Using the same T2DM rat model, our previous study has demonstrated that decreases in N-type Ca^2+^ channels and nicotinic acetylcholine receptors (nAChR) located on CPP neurons contribute to T2DM-attenuated cell excitability of CPP neurons and cardiac vagal activity (Liu et al., 2012, 2015). Reactive oxygen species (ROS) has been reported to modulate the nAChR activation and Ca^2+^ channel kinetics in peripheral postganglionic neurons (Whyte et al., 2009; Krishnaswamy and Cooper, 2012). Reactive oxygen species is overproduced in the T2DM state (Anderson et al., 2009; Henriksen et al., 2011). Therefore, our ongoing study will be focusing on exploring the involvement of ROS in cardiac vagal dysfunction in the T2DM state.

## Conclusion

MI induces fatal ventricular arrhythmias and a high mortality rate in T2DM rats without any effect on CPP neuronal excitability and cardiac vagal function. T2DM caused the withdrawal of cardiac parasympathetic function through decreasing cell excitability of CPP neurons, although T2DM alone does not induce spontaneous ventricular arrhythmias without a high mortality rate. These data indicate that attenuated cardiac parasympathetic function is the main reason for MI-induced fatal ventricular arrhythmia and high mortality rate in the T2DM state. We conclude in the present study that T2DM-reduced CPP neuronal excitability contributes to attenuated cardiac parasympathetic function, which is correlated with MI-induced high mortality rate and malignant ventricular arrhythmias in the T2DM state.

## Data Availability Statement

The original contributions presented in the study are included in the article/Supplementary Material, further inquiries can be directed to the corresponding author.

## Ethics Statement

The animal study was reviewed and approved by the Institutional Animal Care and Use Committee (IACUC) at the University of Nebraska Medical Center.

## Author Contributions

WH, DZ, and Y-LL conceived and designed the experiments and wrote the manuscript. WH, DZ, HT, and Y-LL performed the experiments and analyzed the data. DZ and Y-LL contributed reagents, materials, and analysis tools. All authors contributed to the article and approved the submitted version.

## Conflict of Interest

The authors declare that the research was conducted in the absence of any commercial or financial relationships that could be construed as a potential conflict of interest.

## Publisher’s Note

All claims expressed in this article are solely those of the authors and do not necessarily represent those of their affiliated organizations, or those of the publisher, the editors and the reviewers. Any product that may be evaluated in this article, or claim that may be made by its manufacturer, is not guaranteed or endorsed by the publisher.
