# Supplementary material for: Reduced Cell Excitability of Cardiac Postganglionic Parasympathetic Neurons Correlates With Myocardial Infarction-Induced Fatal Ventricular Arrhythmias in Type 2 Diabetes Mellitus
Source: Front Neurosci. 2021 Aug 18;15:721364. doi: 10.3389/fnins.2021.721364 (PMC8416412; doi:10.3389/fnins.2021.721364)
Supplement: Supplementary file 1 [file Table_1.pdf]

SUPPLEMENTARY MATERIALS TO

Reduced cell excitability of cardiac postganglionic parasympathetic neurons correlates with myocardial infarction-induced fatal ventricular arrhythmias in type 2 diabetes mellitus

Supplemental Table 1. Metabolic characteristics of sham and T2DM rats

	Sham (n=40)	T2DM (n=40)
Body weight (g)	415.7±6.7	384.4±8.4*
Fasting Blood glucose (mg/dl)	93.5±5.8	460.7±11.5*

Data are means ± SEM. Statistical significance was determined by student's unpaired t-test. *P < 0.05 vs. Sham.

Supplemental Table 2. Alterations of electrophysiological properties on APs in CPP neurons from rats with T2DM or MI.

	RMP (mV)	V_{max} (mV/ms)	Overshoot (mV)	APD_{90} (ms)
Sham	-60.9 ± 2.4	147.2 ± 6.6	77.8 ± 2.1	52.7 ± 2.1
T2DM	-60.6 ± 2.6	115.5 ± 3.9*	76.3 ± 2.4	75.6 ± 2.7*
Sham+MI	-60.8 ± 2.5	138.7 ± 4.4	78.3 ± 2.3	57.5 ± 3.4

APs, action potentials; CPP, cardiac parasympathetic postganglionic; T2DM, type 2 diabetes mellitus; MI, myocardial infarction; RMP, resting membrane potential; V_{max}, the maximum rate of depolarization of action potentials; APD_{90}, action potential duration at 90% repolarization. Data are mean ± SEM; n = 10 neurons from 5 rat per group; *$p<0.05$ vs. sham.
